# Effects of genotypes and explants on garlic callus production and endogenous hormones

**DOI:** 10.1038/s41598-020-61564-4

**Published:** 2020-03-17

**Authors:** Hassan H. A. Mostafa, Haiping Wang, Jiangping Song, Xixiang Li

**Affiliations:** 1 Institute of Vegetables and Flowers, Chinese Academy of Agricultural Sciences, The Key Laboratory of Biology and Genetics Improvement of Horticultural Crops, Ministry of Agriculture; 12 Zhongguancun, Nandajie, Haidian District, 100081 Beijing, China; 2 Central Laboratory of Organic Agriculture, Agricultural Research Centre (Affiliation ID: 60019332), 9 Gamaa Street, 12619 Giza, Egypt

**Keywords:** Plant sciences, Plant hormones

## Abstract

High callus production is a feasible way to improve the propagation coefficient of garlic. It remains unknown how genotypes and explants affect garlic callus formation. In the present investigation, we found that there were significant differences in callus formation among garlic varieties. Tip explants were the best calli-producing source, and 91.05% of the explants from four varieties, on average, formed calli after 45 d of primary culturing. Upper leaf parts explants produced lower values. Among the different varieties and explant types, tip explants of variety T141 induced calli in the shortest time and had the greatest callus fresh weight at 45 d. An endogenous hormone contents analysis showed that auxins (indole-3-acetic acid and methyl indole-3-acetic acetate), cytokinins (trans-zeatin and dihydrozeatin), gibberellins_4, 9,15,19,24 and 53_, abscisic acid, jasmonic acid, jasmonoyl-L-isoleucine, and dihydrojasmonic acid were significantly greater in the tips than those in the upper leaf parts. High endogenous jasmonic acid content might play important roles in callus formation. These results will help us not only establish an efficient garlic callus induction protocol that can be applied to large-scale callus multiplication and regeneration, and to genetically improvement of garlic production, but also understand endogenous hormone roles in tissue/organ differentiation and dedifferentiation.

## Introduction

Garlic (*Allium sativum* L.) is an important vegetable crop cultivated worldwide. The commercial varieties of garlic are mainly vegetatively propagated because of its sexual sterility. Consequently, once plants are infected by different viruses, the viruses are transmitted through seed bulbs^1^. In addition, garlic plants in the field have a low propagation rate of approximately 1 to 10 times per year. Therefore, it takes many years to produce a sufficient number of seed bulbs for the practical cultivation of a new variety. Thus, in-vitro tissue culture techniques are feasible alternatives for improving the propagation efficiency, eliminating viruses from infected plants and breeding^2^.

In tissue culture, the callus phase commonly has the objectives of generating genetic variability to obtain new desirable traits and generating transgenic plants to introduce specific traits, such as pest resistance, in *Allium* crops^3–5^. Furthermore, callus production is also a necessary step for obtaining protoplasts used in protoplast fusion, a useful tool in the multiplication and genetic improvement of vegetatively propagated *Allium* species for the production of new varieties^6,7^.

In some *Allium* species, callus induction is difficult, and the proliferation of initial calli is very slow^2,8–10^. Although garlic callus induction has been reported, it is not efficient enough for the rapid mass production of calli^11–14^.

Moreover, *in vitro* cultures are affected by various factors, such as the media, growth regulators, culture conditions, and the explant tissue source^15^. Various garlic tissues have been used to induce calli, such as root-tips, leaves, immature umbels and basal plates, but there are no reports on using the tips and other leaf parts. Exogenous cytokinins and auxins are important in tissue culture to stimulate various developmental and physiological processes^16^. Cytokinins are categorized as being isoprenoid [N6-2-isopentenyl adenine (IP), trans-zeatin (tZ), cis-zeatin (cZ) and dihydrozeatin (DZ)] or aromatic (N6-benzyladenine, kinetin and topolin) types^16^. Abscisic acid (ABA) and jasmonic acid (JA) are phytohormones involved in the tolerance to abiotic stresses^17^. Growth and differentiation in plant tissue culturing are controlled by interactions among exogenous and endogenous hormones^18,19^. The endogenous hormone levels have important effects on the physiological processes, plant architecture^16^ and the initiation of proliferation centers in explants^20^. They are employed frequently to control cell division^17^. Endogenous hormones are involved in the formation and determination of the morphogenic reactions and their concentrations are spatially and temporally regulated in response to external culture conditions^21^. Previous studies have addressed the associations between changes in hormone types and levels and changes in the medium various callus parameters^9,22^. However, most studies only quantified endogenous hormone levels in produced calli. Reports regarding the endogenous hormone levels in explants were evaluated in other plant species i.e. maize^23,24^, wheat^25^, carrot^26^ and *Medicago truncatula* Gaertn^27^. However, the effect of the endogenous hormone contents in garlic explants on callus induction and multiplication is poorly understood.

Consequently, this research aimed to develop an efficient callus induction and proliferation technique for large-scale utilization by comparing callus quantities and growth rates of various explants and genotypes and to understand the callus formation-related roles of endogenous hormones in explants.

## Results

### Number of days for callus emergence in primary cultures

The number of days required for callus induction in four garlic varieties and their different explants was investigated. There were significant differences (*P* < 0.01) among varieties, explants and their interactions regarding the time needed for calli to emerge (Supplementary Material Figure S1). The earliest sign of callus formation was noticeable on an average of five explants of variety T167 after 18 d (Supplementary Material Figure S1a). VarietiesT141 and T167 required shorter times for callus emergence than T36 and CK (25 d) from almost all explants. In addition, the mean number of days for callus emergence was greater in the exterior leaves (25 d on average from all varieties) and upper leaf parts (24 d) (Supplementary Material Figure S1b). The most rapid callus formation was achieved from tip explants (15 d), followed by lower leaf parts (21 d). Additionally, the interaction effects of varieties and explants were also evaluated (Supplementary Material Figure S1c). The results showed that the tip explants of variety T141 started to induce callus after 12 days primary culture, with no significant difference from tips of variety T167 (13 days). In contrast, explants from upper part of leave for variety T36 and exterior leaves of variety CK recorded 32 and 31 days for callus occurrence, respectively.

### The percentage of explants producing calli for the first time

Different varieties and explants, as well as their interactions, significantly affected the percentage of explants producing calli for the first time (Supplementary Material Figure S2). Varieties CK and T36 showed greater average percentages of explants initiating calli (32.66% and 32.58%, respectively) (Supplementary Material Figure S2a). More tip explants, on average, produced calli for the first time (63.57%) than other explants, followed by interior leaves (30.50%). However, upper leaf parts had the lowest mean percentage of explants producing calli for the first time (15.68%) (Supplementary Material Figure S2b). According to the interaction effects of varieties and explant types, the tips of varieties CK and T36 had the greatest percentages of explants that initiated calli (71.87% and 71.67%, respectively). Meanwhile, the upper leaf-part explants from varieties T141, T167 and T36 produced lower percentages calli for the first time, at 14.04%, 15.59% and 15.78%, respectively (Supplementary Material Figure S2c).

### The percentages of total explants producing calli after 45 d of primary culturing

The statistical analysis revealed that there were significantly differences in the percentages of total explants forming calli among garlic varieties. Varieties CK and T36 were superior to the other varieties, with 49.58% and 49.40%, respectively, on average, of all explants forming calli (Supplementary Material Figure S3a). Among explant types, 83.26% of cultured tips produced calli, which was greater than the means of the other five explant types. This was followed by interior leaf explants, with 55.81% of the explants producing calli. The lowest percentage of total explants initiating calli was observed for upper leaf parts at 23.10% from all varieties (Supplementary Material Figure S3b). In addition, the different varietal and explant type combinations significantly affected the percentage of the total explants inducing calli (*P* < 0.01; Supplementary Material Figure S3c). The maximum percentage was noted from tip explants of variety CK (91.05%), but this was not significantly different from the tips of variety T36 (88.00%). The minimum percentage of the total explants producing calli was obtained from upper leaf parts of varieties T141, CK and T167 (21.89%, 22.91% and 23.50%, respectively).

### Callus production after 45 d of primary culturing

**Figure 1:**
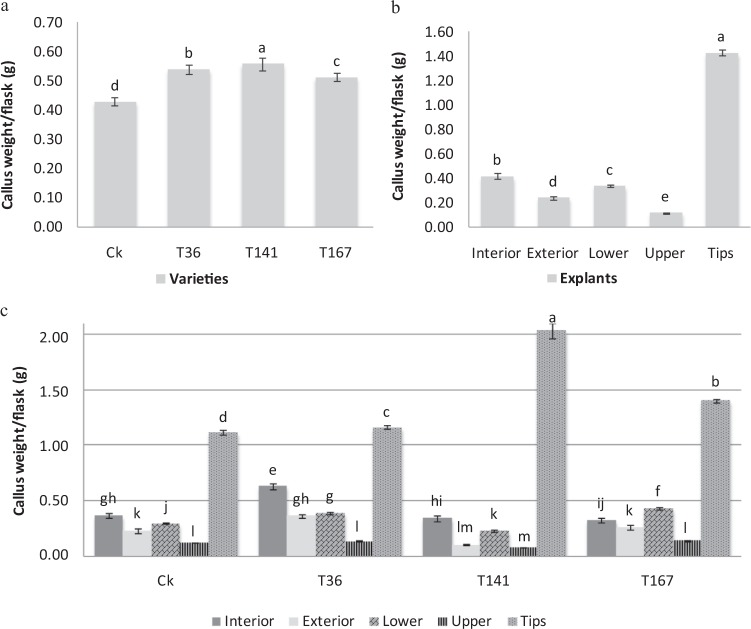
Influence of garlic varieties (**a**), explants (**b**) and their interactions (**c**) on callus weight per flask (g) after 45 d of primary culturing. Data are presented as means ± SDs (n = 3) and the different upper letters indicate significant differences at *P* < 0.05 level according to LSD test.

To determine whether the garlic varieties and explant types affected callus production in primary cultures, the callus weight per flask (g) after 45 d of culturing was measured (Fig. 1). Variety T141 had a significantly greater average callus weight (0.56 g/flask) for all explants compared with the other four varieties. The lowest callus weight was recorded for variety CK (0.43 g/flask) (Fig. 1a). Tips produced the greatest quantity of calli, on average, among all of the garlic varieties (1.43 g/flask), followed by interior leaves (0.42 g/flask). This difference was the great among callus quantities. The upper leaf parts produced the lowest callus weight per flask over the same period (0.12 g; Fig. 1b). The interaction effect between varieties and explants types was highly significant at *P* = 0.01 (Fig. 1c). The tip explants of variety T141 had the greatest callus weight per flask, at 2.04 g, after 45 d. However, callus formation decreased to 0.08 g/flask from upper leaf parts of the same variety.

### Callus cumulative production during subculture

The callus cumulative weight is an important parameter for callus multiplication, somatic embryo induction and plant regeneration. Therefore, the callus weights of four varieties were measured continuously after the 45-d primary induction, during four weeks of subculturing. Supplementary Material Table S1 showed that the effect of explants, genotypes and their interactions on the measured characteristics were highly significant (*P* < 0.01). Variety T36 recorded the greatest callus cumulative weights among all varieties, at 0.89, 1.14, 1.38 and 1.67 g in subculturing W1, W2, W3 and W4, respectively. The lowest cumulative weights were obtained from variety CK, at 0.72, 0.96, 1.17 and 1.40 g/flask for subculturing W1, W2, W3 and W4, respectively. Tip explants produced calli with a significantly greater cumulative weight in all varieties (2.13, 2.65, 3.13 and 3.63 g/flask during subculturing W1, W2, W3 and W4, respectively). This was followed by interior leaves. In contrast, the lowest callus cumulative weight was of calli produced by upper leaf parts in all varieties, at 0.22, 0.30, 0.42 and 0.54 g/flask during subculturing W1, W2, W3 and W4, respectively. Leaf upper parts, in comparison with tip explants, produced 6.74–9.64 times the callus cumulative weight based on all varieties over the 4 weeks. Among the interactions between genotypes and explants, the greatest callus cumulative weight was observed in tip explants of variety T141 during the four weeks of subculturing (3.03, 3.58, 4.34 and 4.99 g/flask forW1, W2, W3 and W4, respectively). However, over the four consecutive weeks of subculturing, the upper leaf part (0.15, 0.20, 0.26 and 0.31 g/flask, respectively) or exterior leaf (0.15, 0.20, 0.24 and 0.30 g/flask, respectively) explants in the same variety (T141) showed the lowest cumulative callus weights.

### Callus growth rates (GRs) in subcultures

The callus growth rate (GR) increases at different culture stages might provide a more concise quantitative characteristic of callus development. The GR value gradually decreased in subcultures of all varieties, and the GR of variety T167 was the greatest during W1 (81.33%), and significantly greater than those of other varieties (Table 1). The GRs for all explant types were greatest during W1 of subculturing and then decreased at different rates. The tip explants had the lowest GR (49.11%) in W1 among all the explants, and their GR continued to gradually decrease over the four weeks (49.11%, 25.43%, 17.69% and 16.54%, respectively). However, the interior leaf and the upper leaf part had the greatest GR values (89.08% and 84.05%, respectively) during W1, but then similarly decreased with time. Interactions between varieties and explants were observed, with the upper leaf part of variety T167 having the greatest GR in W1 (108.57%), while tips had a GR of only 44.01%. Thus, greater callus production rates were obtained in the primary cultures and at the early stages of the subcultures. It appeared that the greater the production, with a greater callus GR, in the primary culture, the lower the production, with a lower callus GR, in the subculture. Thus, undergoing primary culturing and subculturing for proper time lengths might be very important for total callus production.

**Table 1:** The callus growth rate (GR, %) of subculture over primary culture as influenced by garlic varieties and various explants.

Time	Varieties	Interior (±SD)	Exterior (±SD)	Lower (±SD)	Upper (±SD)	Tip (±SD)	Mean
GR1	CK	100.72 ± 6.37^ab^	83.82 ± 4.43^def^	69.56 ± 5.32^hij^	61.83 ± 3.40^jk^	54.04 ± 3.34^kl^	73.99^b^
	T36	81.11 ± 3.37^efg^	84.23 ± 12.56^de^	67.72 ± 4.38^ij^	75.64 ± 5.32^efghi^	49.63 ± 1.80 ^lm^	71.67^b^
	T141	72.68 ± 8.55^ghi^	46.00 ± 5.55 ^lm^	93.14 ± 2.51^bc^	90.14 ± 5.93 ^cd^	48.76 ± 2.15 ^lm^	70.14^b^
	T167	101.79 ± 4.18^ab^	75.02 ± 1.85^fghi^	77.25 ± 2.52^efgh^	108.57 ± 1.55^a^	44.01 ± 0.96 ^m^	81.33^a^
	Mean	89.08^a^	72.11^d^	76.92^c^	84.05^b^	49.11^e^	
GR2	CK	34.44 ± 2.85^de^	41.15 ± 3.89^bc^	30.37 ± 0.88^defg^	47.38 ± 3.58^a^	30.41 ± 2.25^defg^	36.75^a^
	T36	23.98 ± 2.96^gh^	26.97 ± 1.37^fg^	31.54 ± 7.85^def^	31.79 ± 3.05^def^	27.43 ± 1.14^fg^	28.34^b^
	T141	30.49 ± 5.41^defg^	28.49 ± 3.13^efg^	42.05 ± 2.98^ab^	31.46 ± 4.64^def^	18.24 ± 1.40 ^h^	30.14^b^
	T167	24.79 ± 5.45^gh^	29.27 ± 0.95^efg^	35.38 ± 4.45 ^cd^	31.58 ± 8.72^def^	25.64 ± 1.26^fg^	29.33^b^
	Mean	28.42^c^	31.47^b^	34.84^a^	35.55^a^	25.43^d^	
GR3	CK	25.42 ± 0.88^de^	21.86 ± 4.32^ef^	14.72 ± 4.00^gh^	36.51 ± 3.24^b^	20.63 ± 1.09^ef^	23.83^b^
	T36	21.91 ± 2.85^ef^	30.01 ± 3.93^bc^	28.40 ± 3.94 ^cd^	49.33 ± 6.18^a^	9.64 ± 2.29 ^h^	27.86^a^
	T141	20.01 ± 4.30^efg^	20.15 ± 1.8^efg^	32.10 ± 0.64^bc^	31.79 ± 3.89^bc^	21.23 ± 0.64^ef^	25.06^ab^
	T167	20.25 ± 4.19^efg^	18.67 ± 3.13^fg^	22.61 ± 1.22^ef^	36.76 ± 3.75^b^	19.27 ± 1.16^fg^	23.51^b^
	Mean	21.90^b^	22.67^b^	24.46^b^	38.60^a^	17.69^c^	
GR4	CK	22.13 ± 1.22^de^	16.95 ± 4.19^gh^	9.51 ± 1.96^j^	21.90 ± 4.92^defg^	21.73 ± 0.91^defg^	18.45^c^
	T36	22.06 ± 1.45^def^	28.39 ± 3.01^bc^	13.34 ± 5.18^hij^	37.59 ± 6.08^a^	17.62 ± 1.21^efgh^	20.72^a^
	T141	14.74 ± 1.31^hi^	25.68 ± 3.10 ^cd^	15.66 ± 0.37^hi^	20.80 ± 0.12^defg^	14.93 ± 0.35^hi^	18.36^c^
	T167	17.50 ± 2.36^efgh^	17.25 ± 2.71^fgh^	23.78 ± 2.83 ^cd^	33.18 ± 2.90^ab^	11.88 ± 0.44^ij^	20.72^b^
	Mean	19.11^c^	22.06^b^	15.57^d^	28.38^a^	16.54^d^	

Values are given as the mean ± standard deviation (n = 3).

Different letters indicate statistically differences among varieties, explants and their interaction.

### Comparison of endogenous hormone contents in different explants

**Figure 2:**
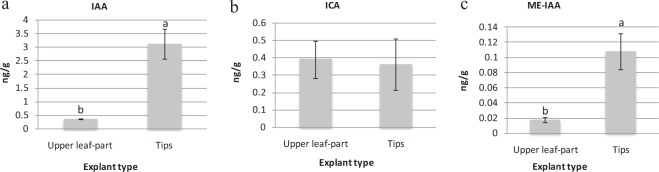
Auxin contents in upper leaf part and tip explants of variety T141: (**a**) indole-3-acetic acid (IAA), (**b**) indole-3-carboxaldehyde (ICA) and (**c**) methyl indole-3-acetate (ME-IAA). Data are presented as means ± SDs (n = 3) and the different upper letters indicate significant differences at *P* < 0.05 level according to LSD test.

Hormone levels were determined for the explants having the greatest callus production (tips) and lowest callus production (upper leaf parts) of variety T141. The endogenous auxin (IAA, ICA and ME-IAA) contents are presented in Fig. 2a–c. No statistically significant differences were found in the ICA contents between these two explant types. However, the IAA level was greatly higher in tip explants (3.12 ng/g) and lower in upper leaf parts (0.35 ng/g). While sharing the same general trend, ME-IAA was much less in the upper leaf parts, at 0.02 ng/g. Additionally, the results of endogenous auxin (IAA and ME-IAA) of varieties Ck, T36 and T167 were in the same trend (Supplementary Material Figure S4).

**Figure 3:**
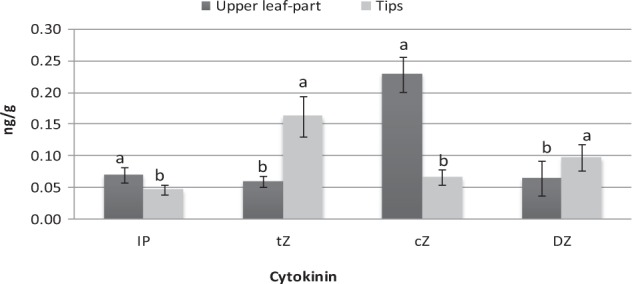
The contents of various endogenous cytokinins (IP, tZ, cZ and DZ) in tips and upper leaf parts of variety T141. Data are presented as means ± SDs (n = 3) and the different upper letters indicate significant differences at *P* < 0.05 level according to LSD test.

Cytokine (IP, cZ, and tZ) concentrations in the two explant types of variety T141 were highly significantly different (Fig. 3). Significantly greater levels of the tZ were found in tip explants, at 0.16 ng/g, than that in upper leaf-part explants. However, upper leaf-part explants had the significantly greater values for IP and cZ hormones at 0.07 ng/g and 0.22 ng/g, respectively.

**Figure 4:**
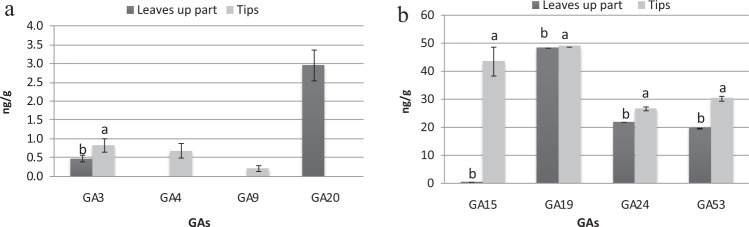
Gibberellin (GA) levels in different explants of variety T141: (**a**) GA3, 4, 9 and 20, (**b**) GA15, 19, 24 and 53. Data are presented as means ± SDs (n = 3) and the different upper letters indicate significant differences at *P* < 0.05 level according to LSD test.

As shown in Fig. 4a,b, there was variation in the GA contents between the different explant types. GA_4_ and GA_9_ were found in tip explants (0.68 ng/g and 0.20 ng/g, respectively) but were not presented in the upper leaf part explants. Same trend was found for other three varieties for GA_9_ (Supplementary Material Figure S5a). In contrast, GA_20_ was found only in upper leaf-part explants, at 2.95 ng/g. In addition, significantly greater concentrations of GA_3, 15, 19, 24 and 53_ were found in tip explants. In particular GA_15, 19, 24 and 53_ were recorded at high values of 43.56, 48.8, 26.63 and 30.19 ng/g, respectively. In the same trend, GA_15_ and GA_24_ were greatly higher in tip explants of varieties CK, T36 and T167 (Supplementary Material Figure S5b,c), although the absolute difference values of various GA components between two kinds of explants were not the same in all varieties.

**Figure 5:**
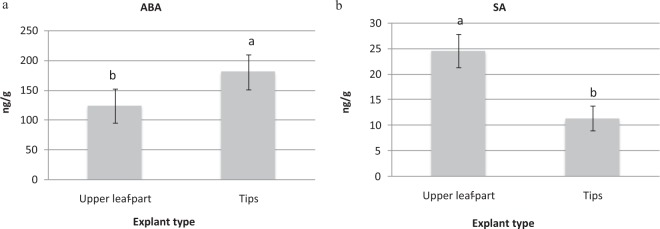
Means of (**a**) abscisic acid (ABA) and (**b**) salicylic acid (SA) concentrations in upper leaf part and tip explants of variety T141. Data are presented as means ± SDs (n = 3) and the different upper letters indicate significant differences at *P* < 0.05 level according to LSD test.

As shown in Fig. 5, differences in the ABA and SA contents in the tips and upper leaf parts of variety T141 were highly significant. For ABA, tip explants produced the maximum value of 180.58 ng/g (Fig. 5a), while upper leaf part explants produced the lower ABA value of 123.89 ng/g. In contrast, a greater SA level was observed in upper leaf part explants (24.708 ng/g), while a lower value (11.40 ng/g) was observed in tip explants (Fig. 5b).

**Figure 6:**
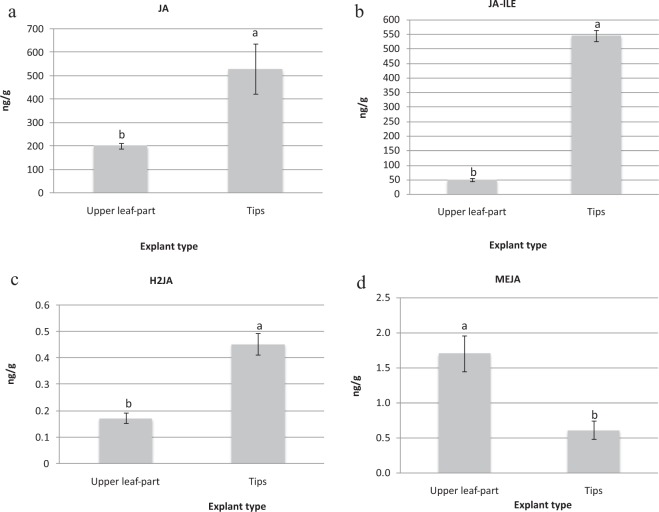
The contents of (**a**) Jasmonic acid (JA), (**b**) jasmonoyl-L-isoleucine (JA-ILE), (**c**) dihydrojasmonic acid (H2JA) and (**d**) methyl jasmonate (MEJA) in upper leaf parts and tips of variety T141. Data are presented as means ± SDs (n = 3) and the different upper letters indicate significant differences at *P* < 0.05 level according to LSD test.

Among the jasmonic hormones, JA, JA-ILE and H2JA showed significantly greater concentrations in the tip explants, at 531.05, 545.14 and 0.45 ng/g, respectively (Fig. 6a–c) than those in upper leaf parts (202.34, 48.51 and 0.17 ng/g). However, the upper part of leaves was higher (1.70 ng/g) than tips (0.60 ng/g) in methyl jasmonate content (Fig. 6d). According to endogenous jasmonic results, the tips of varieties CK, T36 and T167 had the much higher values of JA and JA-ILE than those in the upper part of leaves (Supplementary Material Figure S6).

## Discussion

In tissue culturing, callus induction is an important step in which mature organs dedifferentiate and develop numerous reproducible new plantlets. It is very important for tissue/organ regeneration, massive multiplication and the genetic improvement of plants, especially in vegetative propagated plants.

Our results corroborated previous findings that genotype plays a very important role in callus establishment, growth and subsequent differentiation^24,28–30^. The variations in callus formation and growth among varieties might result from genetic differences. Additionally, Pérez-Jiménez *et al*.^17^ reported that genotypes forming heavier calli might result from the tissues having a greater sensitivity to plant growth regulators.

Furthermore, explant selection is crucial in callus induction and the response of the explant is highly dependent on its genotype and physiological state. Therefore, different types of explants for any given species do not respond equally, resulting in different induction levels of embryogenic calli^31,32^. Here, tip explants produced the most calli during both primary culture and subculture, indicating that they are the best source for rapidly and economically massive producing garlic calli. Our results demonstrated previous reports on Chinese jiaotou^2^. Khar *et al*.^28^ also reported that onion shoot-tip explants are the best source for callus formation. However, Luciani *et al*.^9^ found that basal plates were the best explants for callus differentiation in garlic, but this depends on whether there are shoot-tips remaining on basal plates. Differences in various parameters, such as the numbers of meristematic and parenchymal cells, could be related to the induction of cell division among different explant types, at least when regarding callus growth^33^.

The interaction effects between genotype and explant on callus induction were significant in our experiments. Thus, successful tissue culture and plant regeneration protocols are dependent on plant genotype, explant source and explant developmental stage^34,35^. The tissues’ physiological and metabolic states may be the most important factors for callus production.

Phytohormones are divided into various groups, such as auxins, gibberellins, cytokinins, gaseous compounds (i.e., ethylene), those associated predominantly with growth development and senescence (i.e., ABA), and recently found JA, brassinosteroids and SA^33^. Common exogenous applications of auxins and cytokinins can induce callus formation in various plant species. Also, in some plant species, brassinosteroids and ABA also play important roles in callus induction^36,37^. Although many studies have focused on the reactions of cell and tissue cultures to the supplementation of media with nutrients and phytohormones, investigations into the effects of the endogenous hormonal systems of cultured explants are rather limited^33^. Little experimental evidence is available even though it has been suggested that the endogenous hormone content in the initial explant can affect its capacity to form calli^25,38^. In our study, the endogenous hormone levels of two explant types, tips and upper leaf parts of variety T141, were determined, and the contents of auxins (IAA and ME-IAA), most gibberellins (GA_3, 4, 9, 15, 24 and 53_), cytokinins (tZ), ABA and jasmonic group members JA, JA-ILF etc. were significantly greater in tip explants than those in upper leaf parts. The change trend for most hormones above was similar for other genotypes although the changes and the absolute difference values of some hormones between two kinds of explants were not the same in different genotypes. Growth regulator levels within explants, as endogenous factors, together with the interactions between endogenous and exogenous hormones, may also influence embryogenic responses^30,39^. The level of the endogenous auxin IAA was more than 10 times greater in tip explants than in upper leaf part explants. IAA level is correlated with callus propagation and maintenance^40^. In addition, the results are in accordance with those of Liu *et al*.^41^, in which the promotion of the callus growth in Murashige and Skooge medium supplemented with NAA and kinetin may be the result of the high levels of endogenous IAA and exogenous NAA accumulated in cultured soybean hypocotyl explants. Sasaki *et al*.^42^ and Jiménez and Bangerth^26^, showed that the IAA contents were greater in embryogenic cells than non-embryogenic cells in carrot. In contrast, Michalczuk *et al*.^43^ reported that embryogenic and non-embryogenic callus cells of carrot propagated on 2,4-D-containing medium had similar IAA levels. Additionally, Igielski and Kepczynska^27^ reported that in the *Medicago truncatula* M9–10a line, the amounts of endogenous GAs were probably sufficient for somatic embryogenesis and suggested that the endogenous GA levels may, in some cases, be sufficient for the proliferation of embryogenic calli and embryo development. Likewise, Jiménez and Bangerth^24^ found a significantly greater endogenous GA content in maize embryogenic calli compared with non-embryogenic cells. Our ABA level results supported those of Kiyosue *et al*.^44^, who reported that the endogenous ABA level in embryogenic carrot cells was 67.2 times greater than that in non-embryogenic cells per unit of fresh weight, and Jiménez and Bangerth^26^ found that the ABA level was 10 times greater. In addition, Kiyosue *et al*.^44^ suggested that a high endogenous ABA content might be essential to induce or maintain embryogenic competence in carrot culture systems. The findings of Charrière and Hahne^45^ and Pérez-Jiménez *et al*.^17^ suggested that there is a correlation between morphogenic capacity and endogenous ABA content. Moreover, here, the SA level was lower in the garlic tips than upper leaf parts. This is supported by the results of Quiroz-Figueroa *et al*.^46^, who suggested that a low SA concentration can induce the reprogramming of somatic cells, forcing them into the embryogenic stage, while at a high concentration this reprogramming may be inhibited. This confirmed the correlation between a lower endogenous SA concentration and improved callus induction^47^. Although most previous studies only focused on the hormone levels in the calli produced by tissue culturing, it is evident that greater levels of endogenous JA, IAA and GA hormones in explants are important for garlic callus formation and proliferation. The levels of these endogenous hormones could be used as indicators for the selection of suitable explants for callus production.

The physiological status of the tissues and endogenous hormone levels are of primary importance for tissue dedifferentiation and callus formation. According to Ikeuchi *et al*.^37^, plant meristem is the source of all tissues, and these generative activities are supported by a pool of stem cells residing within the meristem. Thus, the strong stimulation of these meristematic activities leads to ectopic callus induction. Therefore, tender tissues with high endogenous hormone (i.e., JA, IAA and tZ) levels are more suitable for callus induction.

## Conclusion

The effects of genotypes and explant types, as well as their interactions, on garlic callus formation were extremely significant. Stem tip explants were the most suitable, followed by interior leaves, for callus induction. The increase in callus growth rate decreased gradually as the subculture time was extended. Our investigation first demonstrated that greater levels of endogenous auxins (IAA and ME-IAA), gibberellins (GA_3, 4, 9, 15, 24 and 53_) and jasmonic group members (JA, JA-ILF and H2JA) in stem tip explants might play important roles in promoting callus formation and multiplication. These results will help establish a feasible and effective protocol for garlic callus production.

## Materials and Methods

### Plant materials

The plant materials used in the current study between 2016 and 2017 consisted of four garlic varieties from China, Da Xing (CK), T36, T141 and T167, which were provided by the Department of Vegetables Germplasm Resource, Institute of Vegetables and Flowers, Chinese Academy of Agricultural Sciences, Beijing, China.

### Experimental design and basic tissue culture procedure

Different explants of garlic cloves (interior leaves, exterior leaves, lower leaf part, upper leaf part and tips) from the four varieties were used for callus induction (Supplementary Material Figure S7). The experiments were set up in spilt-plot designs, with three replicates per treatment, 10 culture flasks per replication and 10 explants per flask. The main plot was assigned to garlic variety and the sub-plots were assigned to explants.

Healthy cloves were washed with running tap water for 30 min and sterilized by soaking in 75% ethanol for 30 s. This was followed by surface sterilization with 2% sodium hypochlorite solution for 8 to 10 min. After washing three times with sterile distilled water, the explants were detached from the surface-sterilized cloves, cut into 3–4-mm segments and then, cultured on callus induction medium for 45 d.

### Medium and culture condition

The basal medium, containing Murashige and Skooge^48^ nutrients supplemented with 0.5 mg/l naphthalene acetic acid (NAA), 3 mg/l 2,4-dichlorophenoxyactic acid (2,4-D), 0.2 mg/l 6-benzyl amino-adenine, 30 g/l sucrose and solidified with 6 g/l agar, was used as the callus initiation medium. For the weekly callus growth rate (GR) and weekly callus cumulative production, B5^49^ medium (callus propagation medium) supplemented with 1 mg/l 2,4-D, 0.1 mg/l 6-benzyl amino-adenine, 30 g/l sucrose and 6 g/l agar was used. The pH of the medium was adjusted to 5.8 before autoclaving. Calli were incubated at 23 ± 2 °C with a 12/12 h (day/night photoperiod) using cool white fluorescent lamps.

### Data collection for callus induction

The percentage of explants forming calli for the first time was determined once calli emerged from explants cultured in callus induction medium. However, the percentage of total explants producing callus, and the callus weight per flask (g) in primary cultures were measured after 45 d of culturing. Moreover, after 45 d, calli were transferred to a new flask containing 25 ml of callus propagation medium for subculturing, and then, the callus weights were recorded. Over four weeks, the callus cumulative production (g/flask) and callus growth rate (GR) were computed as follows:

$${\rm{GR1}}=\frac{W1-W45d}{W45d}\times 100,$$

$${\rm{GR2}}=\frac{W2-W1}{W1}\times 100,$$

$${\rm{GR3}}=\frac{W3-W2}{W2}\times 100,$$

and

$${\rm{GR4}}=\frac{W4-W3}{W3}\times 100,$$

where W45d represents the callus weight after 45 d of primary culturing, and W1, W2, W3 and W4 represent the callus weights after the first, second, third and fourth weeks of subculturing, respectively.

### Chemicals and regents

HPLC-grade acetonitrile (ACN) and methanol were purchased from Merck (Darmstadt, Germany). MilliQ water (Millipore, Bradford, MA, USA) was used in all experiments. All of the standards were purchased from Olchemim Ltd. (Olomouc, Czech Republic) and Sigma (St. Louis, MO, USA). Acetic acid was purchased from Sinopharm Chemical Reagent (Shanghai, China). The standards’ stock solutions were prepared at 10 mg/ml concentrations in ACN. All stock solutions were stored at −20 °C until used. The stock solutions were diluted with ACN to working solutions before analysis.

### Sample preparation, extraction and analysis

Fresh plant samples were prepared, immediately frozen in liquid nitrogen, and stored at −80 °C until further analyzed. The frozen samples (approximately 120 mg fresh weight) were ground in liquid nitrogen to fine powders by grinding machine (MM 400, Retsch) at 30 Hz for 1 min, homogenized, extracted with 1.2 ml methanol/water (8/2, v/v) and stayed overnight at 4 °C. The extracts were centrifuged at 12,000 g and 4 °C for 15 min. The supernatants were collected, evaporated to dryness under a nitrogen gas stream and reconstituted in methanol/water (3/7, v/v). The solutions were centrifuged, and the supernatants were collected for LC-MS analysis. The hormone measurements were carried out with nine replicates per explant.

The determinations of the contents of auxins (IAA, ME-IAA, indole-3-carboxaldehyde (ICA) and 3-indolebutyric acid); cytokinins (IP, cZ, tZ and DZ); gibberellins (GAs)_1, 3, 4, 7, 9, 15, 19, 20, 24 and 53_, ABA, salicylic acid (SA), methylsalicylate and the jasmonic group (JA, dihydrojasmonic acid (H_2_JA), methyl jasmonate and jasmonoyl-L-isoleucine), were performed using an LC-ESI-MS/MS system, includes Ultra Performance Liquid Chromatography, UPLC (Shim-pack UFLC SHIMADZU CBM30A, http://www.shimadzu.com.cn/) and Tandem mass spectrometry, MS/MS (Applied Biosystems 6500 Quadrupole Trap, http://www.appliedbiosystems.com.cn/) by Wuhan Metware Biotechnology Co., Ltd., Wuhan, China^50–52^.

### HPLC conditions

The HPLC analytical conditions were as follows: HPLC column, Waters ACQUITY UPLC HSS T3 C18 (1.8 µm, 2.1 mm × 100 mm); solvent system, water (0.04% acetic acid): acetonitrile (0.04% acetic acid); gradient program, 95:5 V/V at 0 min, 5:95 V/V at 11.0 min, 5:95 V/V at 12.0 min, 95:5 V/V at 12.1 min and 95:5 V/V at 15.0 min; flow rate, 0.35 ml/min; temperature, 40 °C; and injection volume: 5 μl. The effluent was connected to an ESI-triple quadrupole-linear ion trap (Q TRAP)-MS.

### ESI-Q TRAP-MS/MS

An API 6500 Q TRAP LC/MS/MS System, equipped with an ESI Turbo Ion-Spray interface, operating in a positive ion mode and controlled by Analyst 1.6 software (AB Sciex), was used. The ESI source operation parameters were as follows: ion source, turbo spray; source temperature, 500 °C; ion spray voltage, 5,500 V; curtain gas set at 35.0 psi; and the collision gas was medium. DP and CE values for individual MRM transitions were established by further optimization. A specific set of MRM transitions were monitored for each period based on the plant hormones eluted within the period.

### Hormone qualitative and quantitative analysis

MWDB (metware database) plant hormone database was constructed based on plant hormone standard products, and then the data of mass spectrometry were analyzed qualitatively to characterize a substance according to its ion pair information (Q1, Q3, RT, DP and CE) (Supplementary Material Table S2). Hormone quantification was analyzed using multiple reaction monitoring (MRM) of triple quadrupole mass spectrometry system. The software Analyst 1.6.1 was used to process the mass spectrum data. Total ions current (TIC) and count per second (cps) were obtained (Supplementary Material Figure S8). The repeatability of hormone extraction and detection can be judged by overlapping display analysis of the total ion flow diagrams (TIC diagrams) of different quality control samples. According to the information of hormone retention time and peak pattern, the mass spectrum peaks detected in different samples for each hormone were corrected to ensure the accuracy of qualitative and quantitative analysis. The peak area of each chromatographic peak represented the relative content of corresponding hormones, and the results of quantitative analysis of all samples of hormones were finally obtained based on the standard curve of external standard method.

Plant hormone standard solutions with different concentrations of 0.02 ng/mL, 0.05 ng/mL, 0.1 ng/mL, 0.2 ng/mL, 0.5 ng/mL, 1 ng/mL, 2 ng/mL, 5 ng/mL, 10 ng/mL, 20 ng/mL, 30 ng/mL, 50 ng/mL, 100 ng/mL, 200 ng/mL, 400 ng/mL, 1600 ng/mL and 2000 ng/mL were prepared. Obtain the peak intensity data of mass spectrum corresponding to the quantitative signal of each standard concentration; Standard curves of different hormones were plotted (Supplementary Material Figures S9–13).

The integral peak area of all detected hormones was substituted into the linear equations of the standard curves for calculation, and then the absolute content of hormones in the actual samples were obtained after further calculation as follows:

$${\rm{The}}\,{\rm{content}}\,{\rm{of}}\,{\rm{hormone}}\,{\rm{in}}\,{\rm{the}}\,{\rm{sample}}\,({\rm{ng}}/{\rm{g}})={\rm{B}}\ast {\rm{C}}/1000/{\rm{D}}\ast {\rm{E}}/{\rm{F}}/{\rm{g}}$$

Meaning of each letter in the formula:

B: concentration value (ng/mL) obtained by substituting the peak area of hormone integral in the sample into the standard curve;

C: volume of solution used in redissolution (100 mul);

D: volume of supernatant collected during sample extraction (1100 micron);

E: volume (mul) of extracted solution added during sample extraction;

F: standard recovery rate of hormone addition (%);

G: weigh the sample mass (g).

### Statistical analyses

An analysis of variance to determine the influences of different varieties, explants and their interactions was done using Statistix8.1 software^53^. A means separation was performed using the least significant difference test at *P* = 0.05. Data are presented as the means ± standard deviations.

## Supplementary information

Supplementary Information.
