# Supplementary material for: Effects of genotypes and explants on garlic callus production and endogenous hormones
Source: Sci Rep. 2020 Mar 17;10:4867. doi: 10.1038/s41598-020-61564-4 (PMC7078195; doi:10.1038/s41598-020-61564-4)
Supplement: Supplementary file 1 — Supplementary Information. [file 41598_2020_61564_MOESM1_ESM.pdf]

Supplementary Information

Effects of genotypes and explants on garlic callus production and endogenous hormones

Hassan H.A. Mostafa[1, 2], Wang Haiping[1], Song Jiangping[1] and Li Xixiang[1,*]

[1]Institute of Vegetables and Flowers, Chinese Academy of Agricultural Sciences; The Key Laboratory of Biology and Genetics Improvement of Horticultural Crops, Ministry of Agriculture; 12 Zhongguancun, Nandajie, Haidian District, 100081Beijing, China.

[2]Central Laboratory of Organic Agriculture, Agricultural Research Centre (Affiliation ID: 60019332), 9 Gamaa Street, 12619 Giza, Egypt

[*]Corresponding Author: E-mail address: lixixiang@caas.cn

Supplementary Material Figure S1. Influence of garlic varieties (a), explants (b) and the interaction between varieties and explants (c) on the number of days for callus emergence in primary cultures. Data are presented as means ± SDs (n=3) and the different upper letters indicate significant differences at $P< 0.05$ level according to LSD test.

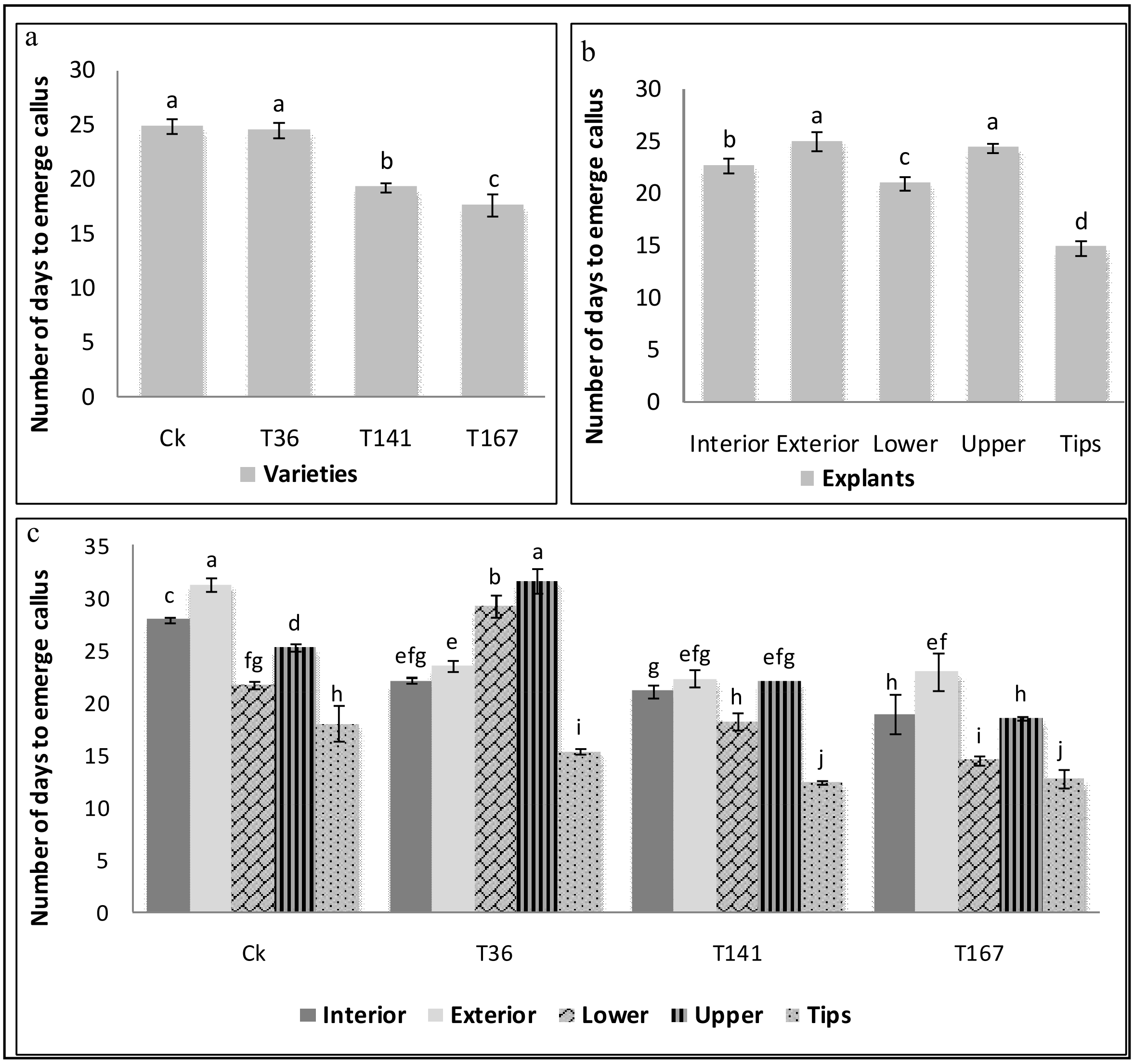

Supplementary Material Figure S2. Effects of garlic varieties (a), explants (b) and their interaction on the percentage of explants producing calli for the first time. Data are presented as means ± SDs (n=3) and the different upper letters indicate significant differences at $P < 0.05$ level according to LSD test.

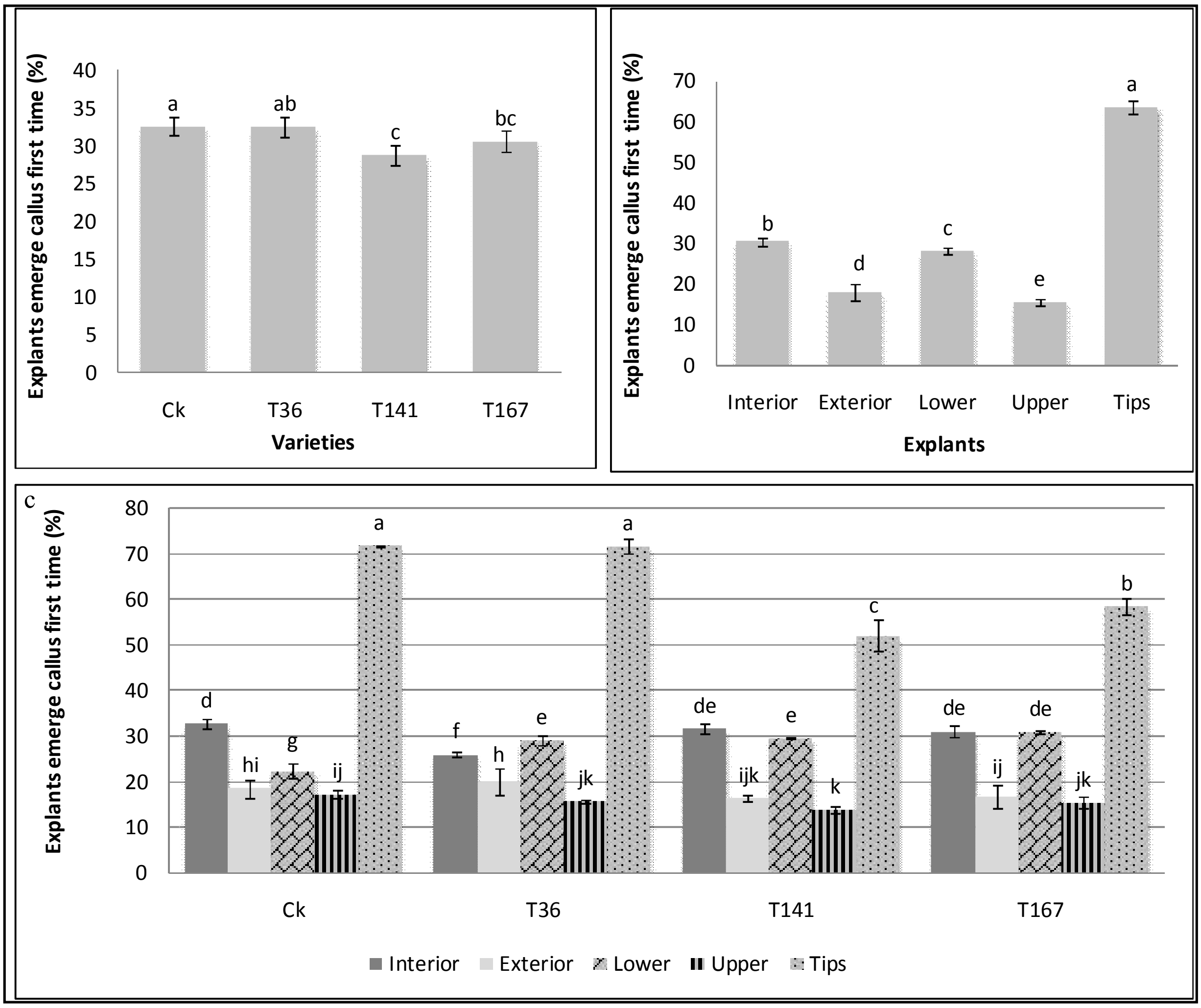

Supplementary Material Figure S3. Percentage of total explants producing calli after 45 days of primary culturing as influenced by (a) garlic varieties, (b) explants and (c) their interaction. Data are presented as means ± SDs (n=3) and the different upper letters indicate significant differences at $P< 0.05$ level according to LSD test.

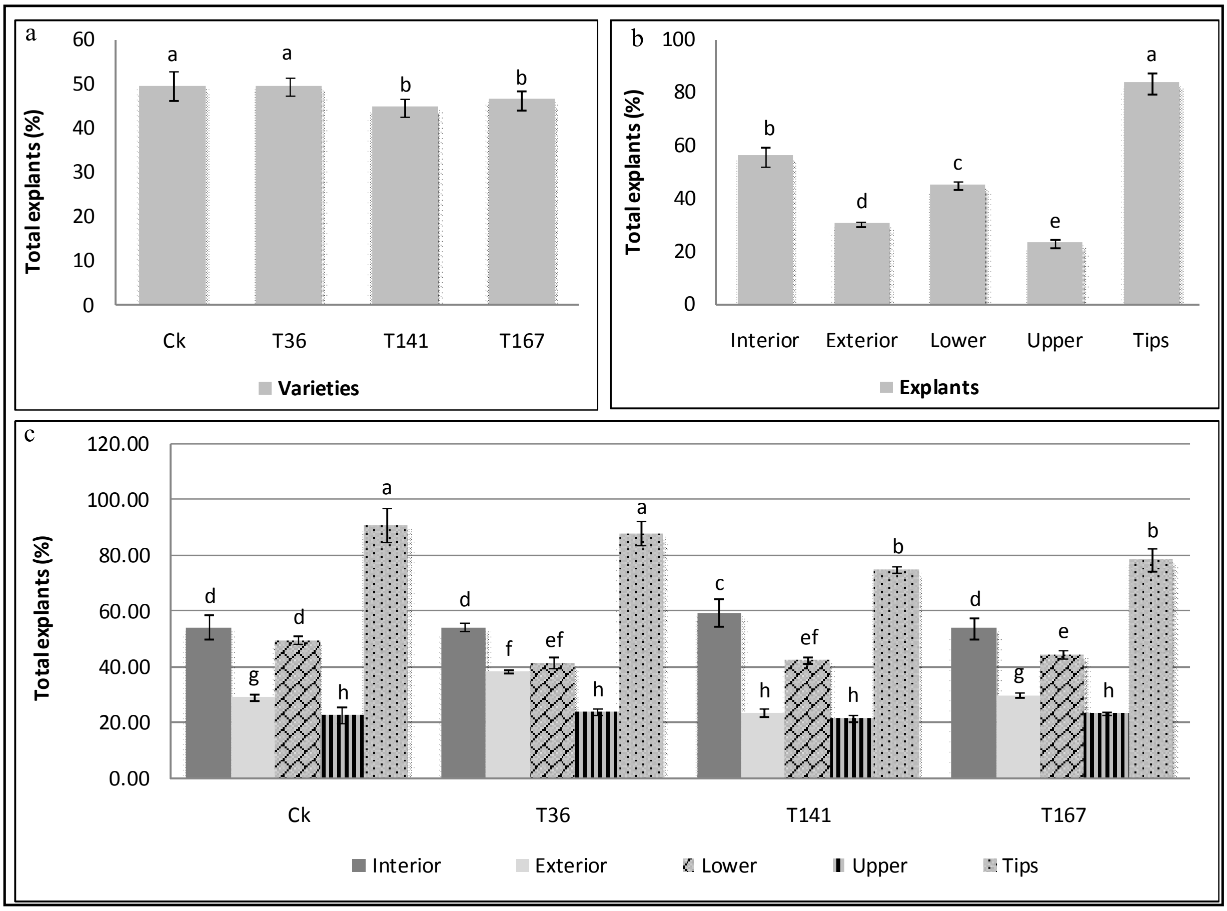

Supplementary Material Figure S4. Auxin contents in upper leaf part and tip explants of varieties CK, T36 and T167: (a) indole-3-acetic acid (IAA) and (b) methyl indole-3-acetate (ME-IAA). Data are presented as means ± SDs (n=3) and the different upper letters indicate significant differences at *P*< 0.05 level according to LSD test.

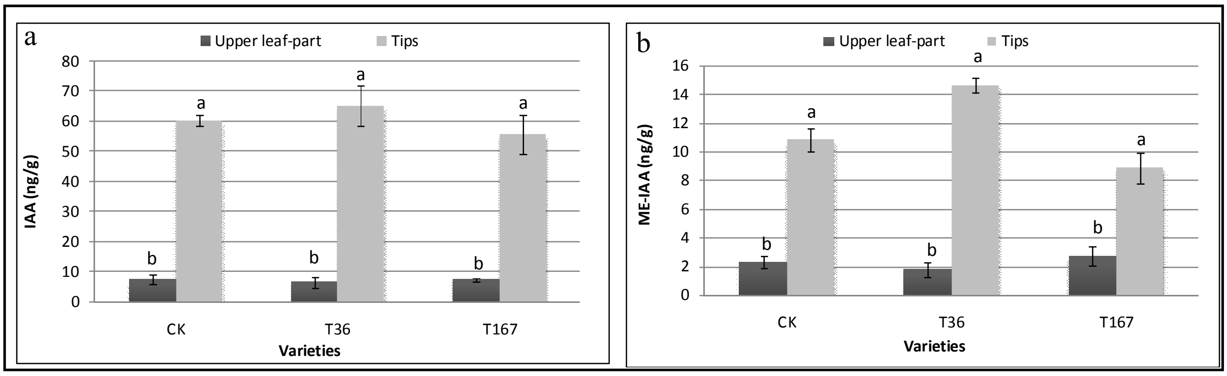

Supplementary Material Figure S5. Gibberellin (GA) levels in different explants of varieties CK, T36 and T167: (a) GA9, GA15 and GA24. Data are presented as means ± SDs (n=3) and the different upper letters indicate significant differences at $P < 0.05$ level according to LSD test.

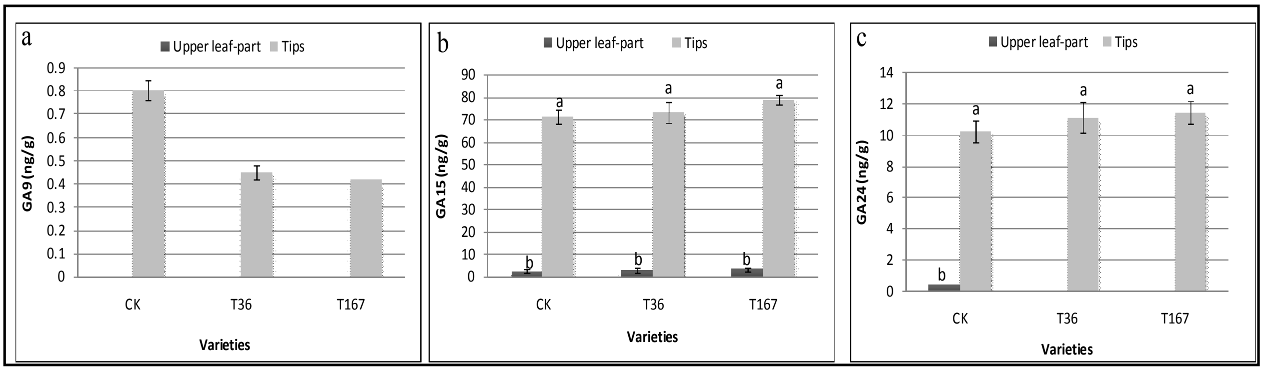

Supplementary Material Figure S6. The contents of (a) Jasmonic acid (JA) and (b) jasmonoyl-L-isoleucine (JA-ILE) in upper leaf parts and tips of varieties CK, T36 and T167. Data are presented as means ± SDs (n=3) and the different upper letters indicate significant differences at *P*< 0.05 level according to LSD test.

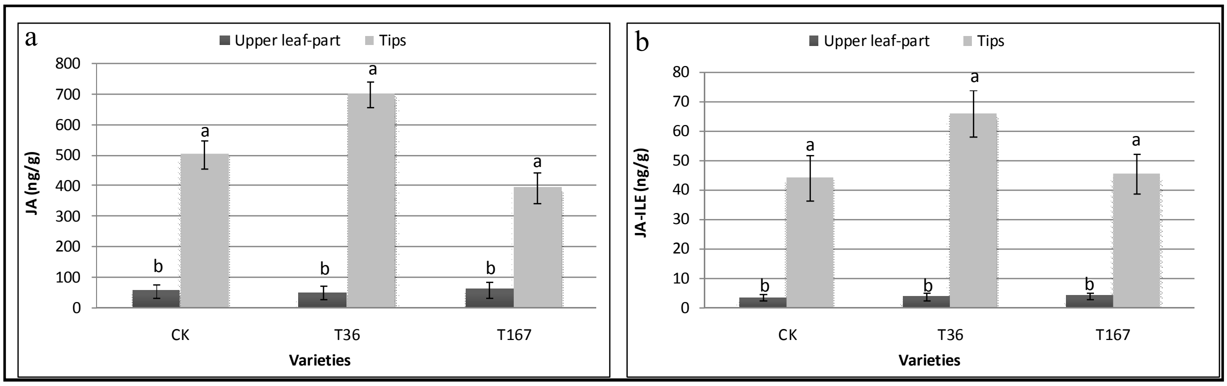

Supplementary Material Figure S7. Garlic explants; interior leaves, exterior leaves, lower leaf-part, upper leaf part, and tips.

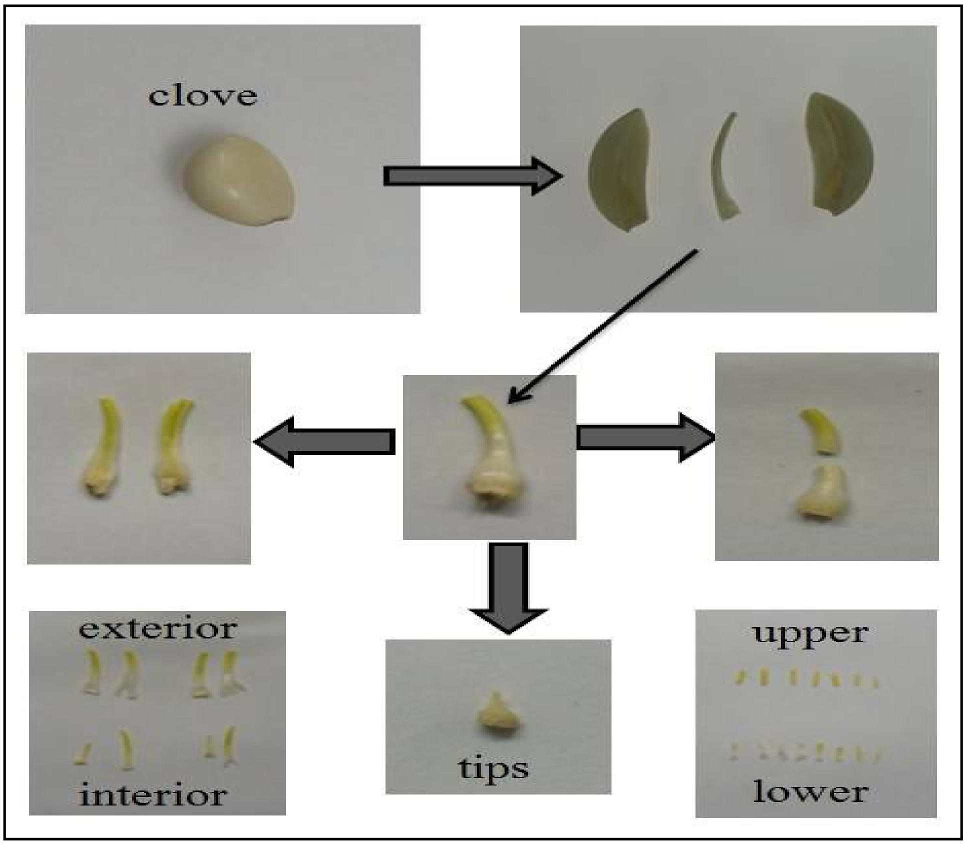

Supplementary Material Figure S8. Total ions current (TIC) of samples. X-coordinate denotes Retention time, Rt; Y-coordinate represents count per second (cps)

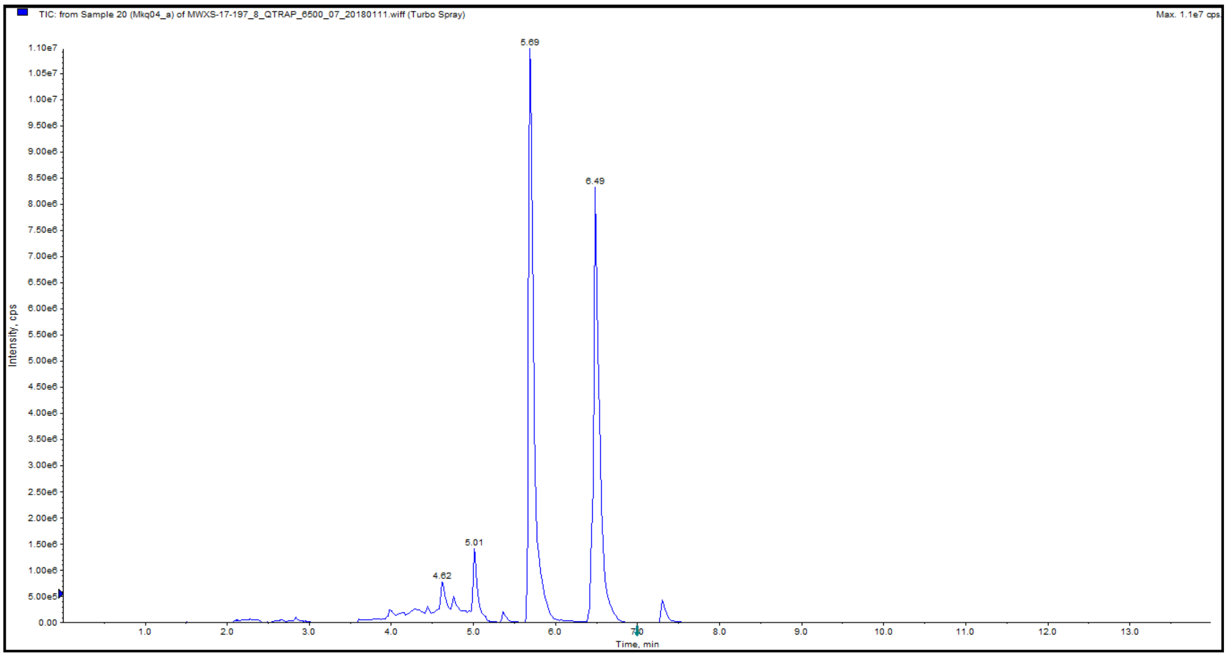

Supplementary Material Figure S9. Standard curves of auxins: (a) indole-3-acetic acid (IAA), (b) indole-3-carboxaldehyde (ICA) and (c) methyl indole-3-acetate (ME-IAA).

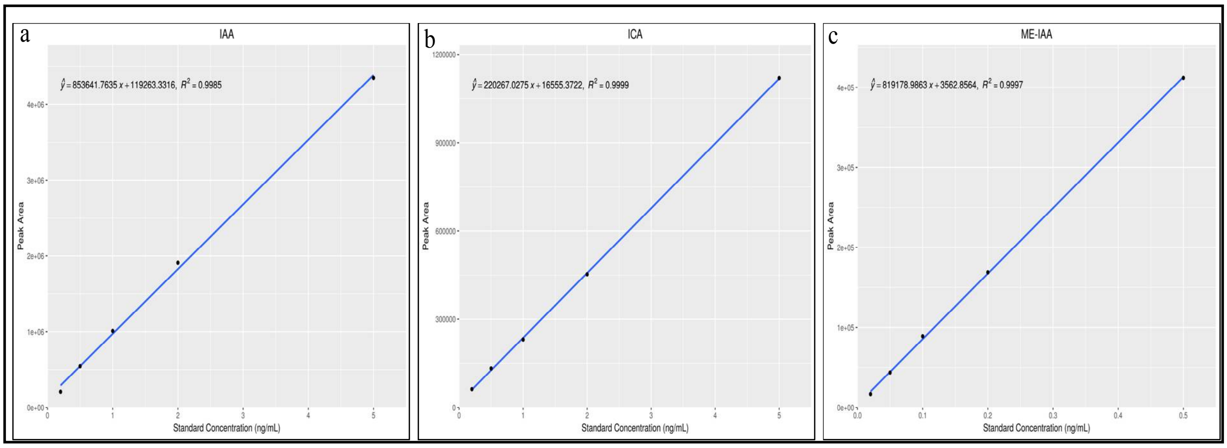

Supplementary Material Figure S10. Standard curves of cytokinins (IP, tZ, cZ and DZ)

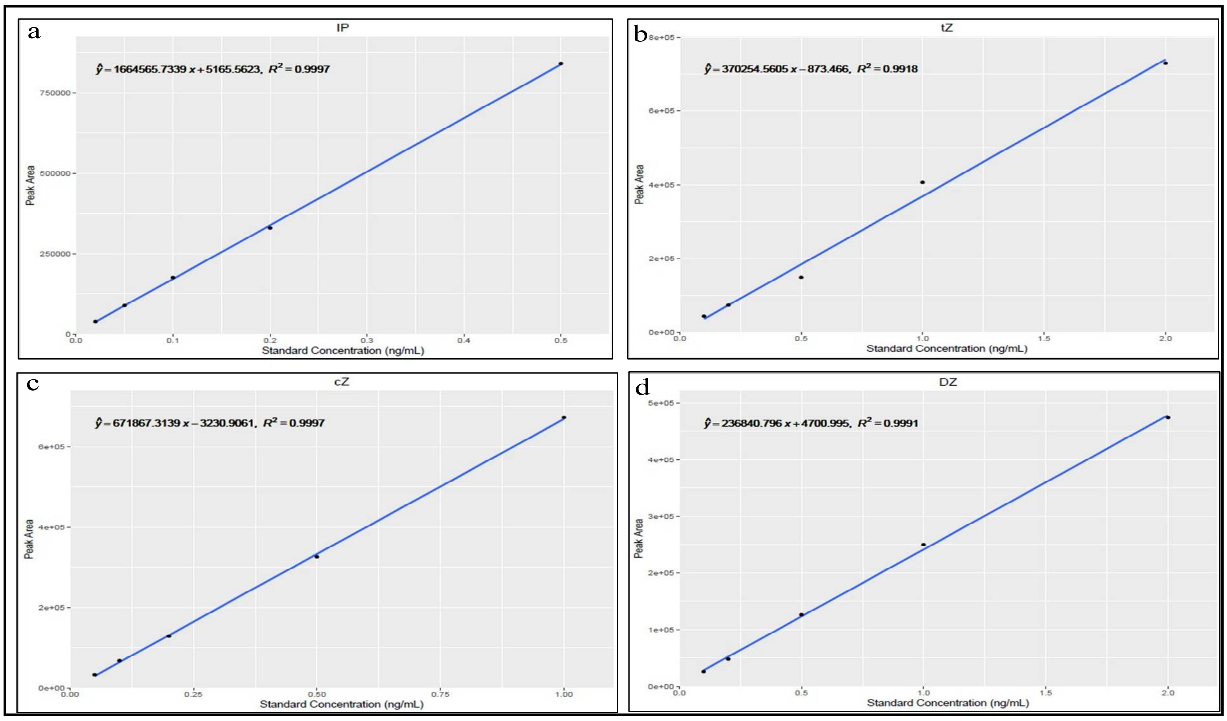

Supplementary Material Figure S11. Standard curves of gibberellins: (a) GA_3, (b) GA_4, (c) GA_9, (d) GA_{15}, (e) GA_{19}, (f) GA_{20}, (g) GA_{24} and (h) GA_{53}.

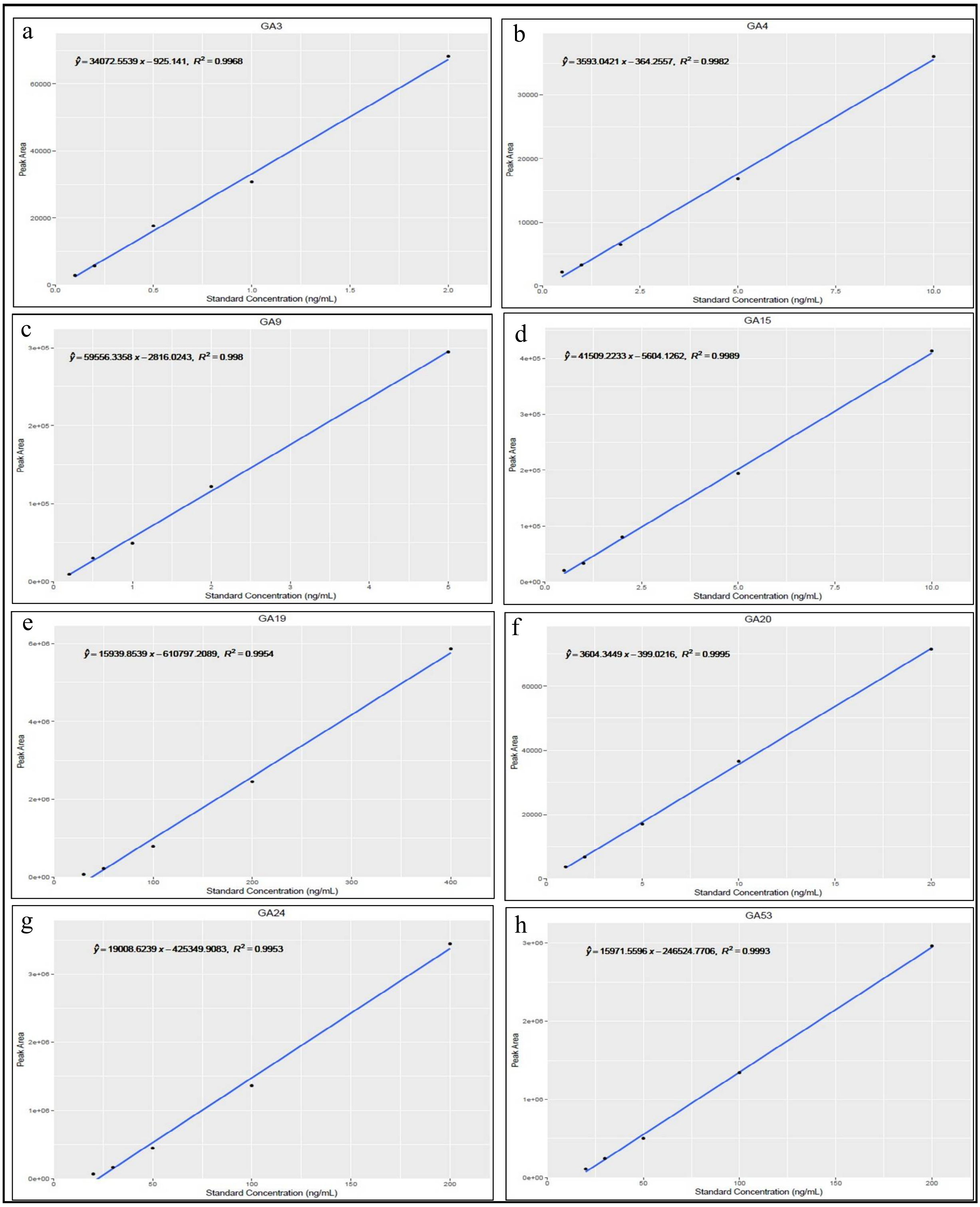

Supplementary Material Figure S12. Standard curves of (a) abscisic acid (ABA) and (b) salicylic acid (SA).

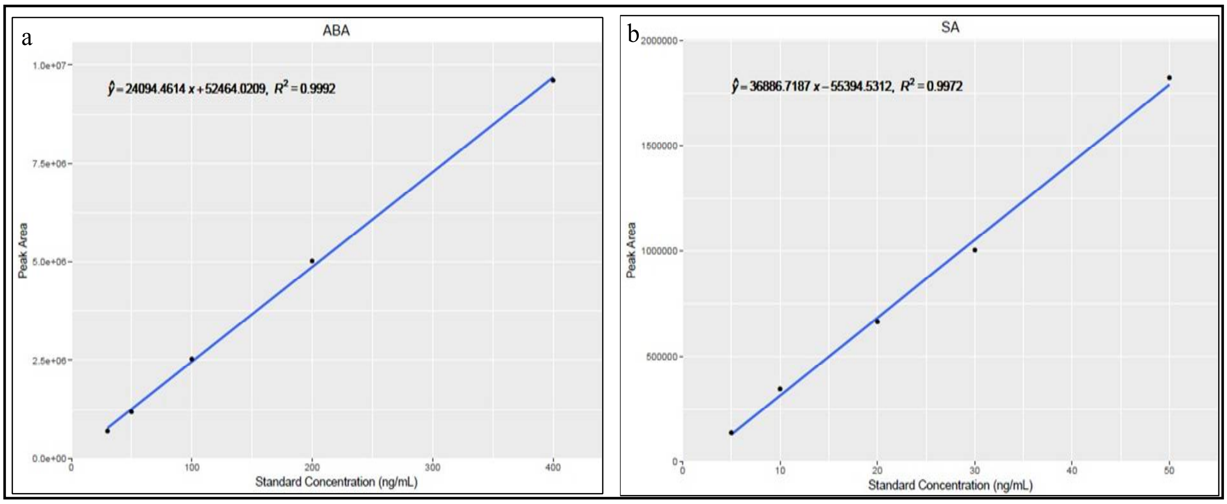

Supplementary Material Figure S13. Standard curves of Jasmonic group: (a) Jasmonic acid (JA), (b) jasmonoyl-L-isoleucine (JA-ILE), (c) dihydrojasmonic acid (H2JA) and (d) methyl jasmonate (MEJA).

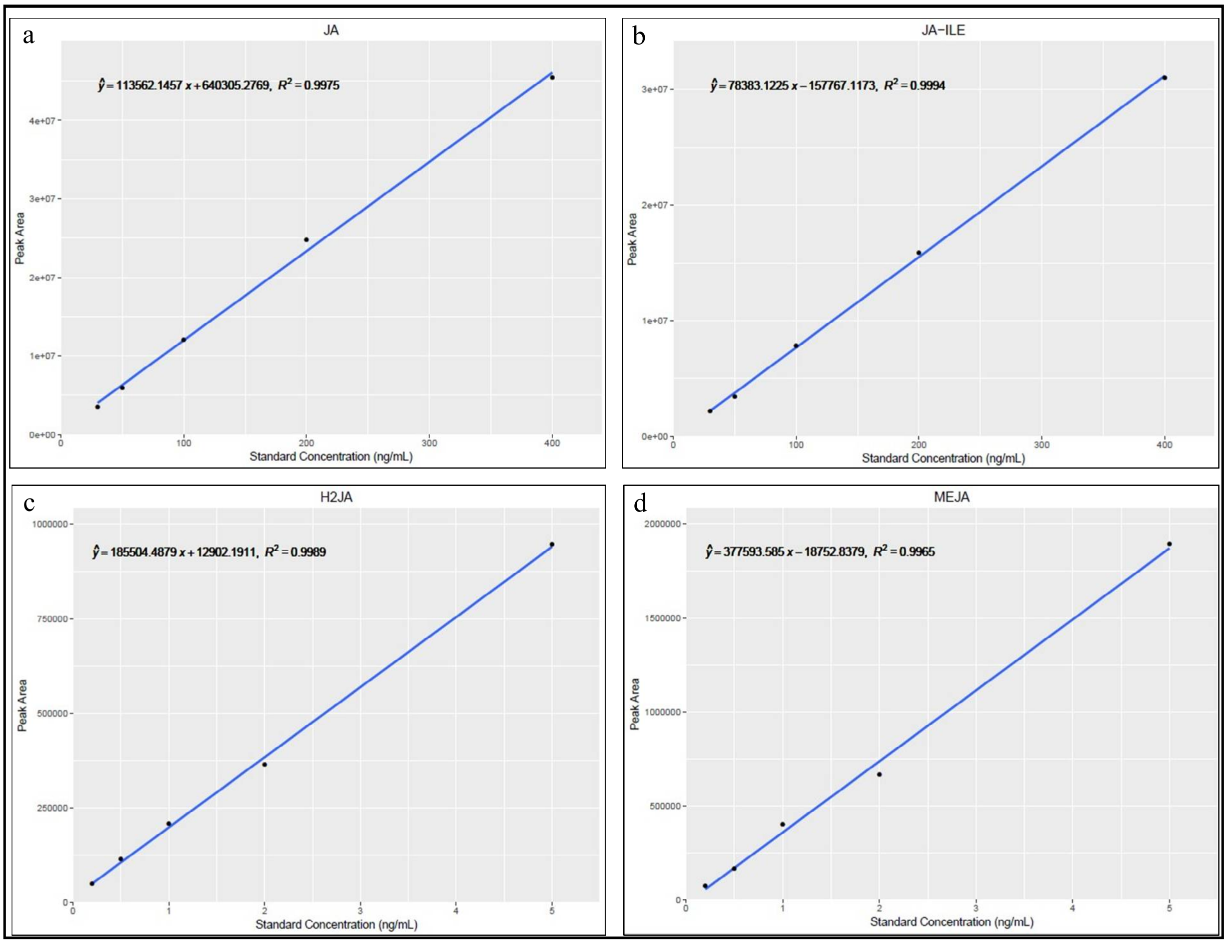

Supplementary Material Table S1. Effect of explants, genotypes and their interactions on callus cumulative production (g/flask) during subculture

Time	Varieties	Interior (±SD)	Exterior (±SD)	Lower (±SD)	Upper (±SD)	Tip (±SD)	Mean
First week of subculture	CK	0.749 ± 0.07^{e}	0.430 ± 0.05^{i}	0.502 ± 0.02^{h}	0.199 ± 0.00^{kl}	1.723 ± 0.02^{c}	0.721^{d}
	T36	1.149 ± 0.05^{d}	0.675 ± 0.04^{f}	0.662 ± 0.02^{f}	0.243 ± 0.01^{jk}	1.747 ± 0.04^{c}	0.895^{a}
	T141	0.597 ± 0.02^{g}	0.155 ± 0.01^{l}	0.439 ± 0.02^{i}	0.149 ± 0.01^{l}	3.028 ± 0.06^{a}	0.874^{b}
	T167	0.656 ± 0.05^{f}	0.454 ± 0.04^{hi}	0.772 ± 0.01^{e}	0.293 ± 0.01^{j}	2.022 ± 0.03^{b}	0.839^{c}
	Mean	0.788^{b}	0.428^{d}	0.594^{c}	0.221^{e}	2.130^{a}	
Second week of subculture	CK	1.006 ± 0.09^{e}	0.608 ± 0.08^{h}	0.654 ± 0.03^{h}	0.293 ± 0.01^{j}	2.248 ± 0.07^{c}	0.962^{c}
	T36	1.425 ± 0.08^{d}	0.857 ± 0.05^{fg}	0.871 ± 0.05^{f}	0.320 ± 0.01^{ij}	2.227 ± 0.06^{c}	1.140^{a}
	T141	0.779 ± 0.03^{g}	0.199 ± 0.01^{k}	0.623 ± 0.02^{h}	0.196 ± 0.00^{k}	3.580 ± 0.02^{a}	1.075^{b}
	T167	0.818 ± 0.06^{fg}	0.587 ± 0.05^{h}	1.045 ± 0.03^{e}	0.385 ± 0.01^{i}	2.540 ± 0.02^{b}	1.075^{b}
	Mean	1.007^{b}	0.563^{d}	0.798^{c}	0.299^{e}	2.649^{a}	
Third week of subculture	CK	1.263 ± 0.12^{f}	0.741 ± 0.10^{ij}	0.750 ± 0.01^{ij}	0.400 ± 0.01^{l}	2.712 ± 0.10^{c}	1.173^{c}
	T36	1.737 ± 0.10^{e}	1.115 ± 0.10^{g}	1.117 ± 0.03^{g}	0.477 ± 0.02^{kl}	2.441 ± 0.09^{d}	1.377^{a}
	T141	0.935 ± 0.06^{h}	0.239 ± 0.00^{m}	0.823 ± 0.02^{i}	0.258 ± 0.01^{m}	4.340 ± 0.05^{a}	1.319^{ab}
	T167	0.984 ± 0.08^{h}	0.698 ± 0.08^{j}	1.281 ± 0.02^{f}	0.527 ± 0.01^{k}	3.030 ± 0.04^{b}	1.304^{b}
	Mean	1.230^{b}	0.698^{d}	0.993^{c}	0.416^{e}	3.131^{a}	
Fourth week of subculture	CK	1.543 ± 0.16^{e}	0.869 ± 0.15^{i}	0.821 ± 0.01^{ij}	0.488 ± 0.01^{l}	3.301 ± 0.10^{b}	1.404^{c}
	T36	2.121 ± 0.15^{d}	1.433 ± 0.15^{e}	1.265 ± 0.03^{f}	0.657 ± 0.05^{jk}	2.871 ± 0.10^{c}	1.670^{a}
	T141	1.073 ± 0.08^{gh}	0.300 ± 0.01^{m}	0.952 ± 0.03^{hi}	0.312 ± 0.02^{m}	4.988 ± 0.04^{a}	1.525^{b}
	T167	1.157 ± 0.11^{fg}	0.819 ± 0.10^{ij}	1.585 ± 0.03^{e}	0.701 ± 0.01^{jk}	3.390 ± 0.04^{b}	1.531^{b}
	Mean	1.474^{b}	0.855^{d}	1.156^{c}	0.540^{e}	3.638^{a}	

Values are given as the mean ± standard deviation (n=3).
Different letters indicate statistically differences among varieties, explants and their interaction.

Supplementary Material Table S2. Plant hormone ion pairs, detection limits and recovery rates.

Index	Molecular Weight (Da)	Ion mode	Ionization model	Q1 (Da)	Q3 (Da)	Rt (min)	Compounds	Abbreviat-ion	Category	KEGG ID	Instrument detection limit (ng/g)	Recovery (%)
1	175.18	Positive	[M+H]+	176.1	130.1	4.65	Indole-3-acetic acid	IAA	Auxin	C00954	0.0009	80
2	145.16	Positive	[M+H]+	146.1	91	4.47	Indole-3-carboxaldehyde	ICA	Auxin	C08493	0.0004	80
3	145.16	Positive	[M+H]+	146.1	117	4.47	Indole-3-carboxaldehyde	ICA_1	Auxin	C08493	0.0017	80
4	189.21	Positive	[M+H]+	190.1	130	6.12	Methyl indole-3-acetate	ME-IAA	Auxin	-	0.0007	70
5	189.21	Positive	[M+H]+	190.1	103	6.12	Methyl indole-3-acetate	ME-IAA_1	Auxin	-	0.0097	70
6	203.24	Negative	[M-H]-	202.1	158	5.71	3-Indolebutyric acid	IBA	Auxin	C11284	0.0103	80
7	203.24	Negative	[M-H]-	202.1	184	5.71	3-Indolebutyric acid	IBA_1	Auxin		0.0996	80
8	203.24	Positive	[M+H]+	204.25	136	3.93	N6-Isopentenyladenine	IP	CK	-	0.0003	70
9	203.24	Positive	[M+H]+	204.25	118.9	3.93	N6-Isopentenyladenine	IP_1	CK	-	0.0023	70
10	219.24	Positive	[M+H]+	220.25	136.1	2.51	trans-Zeatin	tZ	CK	C15545	0.0023	90
11	219.24	Positive	[M+H]+	220.25	136.1	2.67	cis-Zeatin	cZ	CK	C15545	0.0011	90
12	219.24	Positive	[M+H]+	220.25	119.2	2.67	cis-Zeatin	cZ_1	CK	C15545	0.0083	90
13	221.26	Positive	[M+H]+	222.26	136.1	2.57	Dihydrozeatin	DZ	CK	C02029	0.0027	90
14	221.26	Positive	[M+H]+	222.26	147.9	2.57	Dihydrozeatin	DZ_1	CK	C02029	0.0133	90
15	348.39	Negative	[M-H]-	347.4	241	3.92	Gibberellin A1	GA1	GA	C00859	0.0723	78
16	346.37	Negative	[M-H]-	345.01	239	3.88	Gibberellin A3	GA3	GA	C01699	0.018	78
17	346.37	Negative	[M-H]-	345.01	143	3.88	Gibberellin A3	GA3_1	GA	C01699	0.0139	78
18	332.39	Negative	[M-H]-	331.4	212.9	6.27	Gibberellin A4	GA4	GA	C11864	0.0863	85
19	330.37	Negative	[M-H]-	329.01	222.7	6.17	Gibberellin A7					
20	316.39	Negative	[M-H]-	315.2	271.2	7.38	Gibberellin A9	GA9	GA	C11863	0.0081	78
21	316.39	Negative	[M-H]-	315.2	253.1	7.38	Gibberellin A9	GA9_1	GA	C11863	0.0432	78
22	330.41	Negative	[M-H]-	329.2	257	7.36	Gibberellin A15	GA15	GA	C14162	0.0095	82
23	362.41	Negative	[M-H]-	361.2	317	4.9	Gibberellin A19	GA19	GA	C02034	1.1261	73
24	332.39	Negative	[M-H]-	331.2	287	5.11	Gibberellin A20	GA20	GA	C02035	0.019	83
25	332.39	Negative	[M-H]-	331.2	243	5.11	Gibberellin A20	GA20_1	GA	C02035	0.1292	83
26	346.41	Negative	[M-H]-	345.2	257.2	6.54	Gibberellin A24	GA24	GA	C11861	0.5669	94
27	346.41	Negative	[M-H]-	345.2	301.1	6.54	Gibberellin A24	GA24_1	GA	C11861	0.3333	94
28	348.43	Negative	[M-H]-	347.1	329.1	5.73	Gibberellin A53	GA53	GA	C06094	0.1298	73
29	264.32	Negative	[M-H]-	263	219	5.05	(+)-cis,trans-Abscisic acid	ABA	ABA	C06082	0.0163	90
30	264.32	Negative	[M-H]-	263	203	5.05	(+)-cis,trans-Abscisic acid	ABA_1	ABA	C06082	0.0514	90
31	138.12	Negative	[M-H]-	137	93	4.11	Salicylic acid	SA	SA	C00805	0.1444	90

32	138.12	Negative	[M-H]-	137	65	4.11	Salicylic acid	SA_1	SA	C00805	0.0142	90
33	152.15	Positive	[M+H]+	153	121	6.81	Methylsalicylate	MESA	SA	C14088	0.0849	80
34	210.27	Negative	[M-H]-	209	59	5.75	(+)-Jasmonic acid	JA	JA	C08491	0.0054	90
35	210.27	Negative	[M-H]-	209	41	5.75	(+)-Jasmonic acid	JA_1	JA	C08491	0.0464	90
36	323.43	Negative	[M-H]-	322.40	129.7	6.51	N-[(-)-Jasmonoyl]-(L)-Isoleucine	JA-ILE	JA	C18699	0.0203	90
37	212.29	Negative	[M-H]-	211.1	59	6.25	(+)-Dihydrojasmonic acid	H2JA	JA	-	0.0019	80
38	212.29	Negative	[M-H]-	211.1	167	6.25	(+)-Dihydrojasmonic acid	H2JA_1	JA	-	0.1225	80
39	224.29	Positive	[M+H]+	225.1	151	7.43	Methyl jasmonate	MEJA	JA	C11512	0.0017	80
